# Tetrathiafulvalenes as anchors for building highly conductive and mechanically tunable molecular junctions

**DOI:** 10.1038/s41467-022-29483-2

**Published:** 2022-04-04

**Authors:** Qi Zhou, Kai Song, Guanxin Zhang, Xuwei Song, Junfeng Lin, Yaping Zang, Deqing Zhang, Daoben Zhu

**Affiliations:** 1 Beijing National Laboratory for Molecular Sciences, CAS Key Laboratory of Organic Solids, Institute of Chemistry, Chinese Academy of Sciences, 100190 Beijing, China; 2 University of Chinese Academy of Sciences, 100049 Beijing, China

**Keywords:** Molecular electronics, Electronic materials

## Abstract

The interface between molecules and electrodes has great impact on charge transport of molecular devices. Precisely manipulating the structure and electronic coupling of electrode-molecule interface at a molecular level is very challenging. Here, we develop new molecular junctions based on tetrathiafulvalene (TTF)-fused naphthalene diimide (NDI) molecules which are anchored to gold electrodes through direct TTF-Au contacts formed via Au-S bonding. These contacts enable highly efficient orbital hybridization of gold electrodes and the conducting π-channels, yielding strong electrode-molecule coupling and remarkably high conductivity in the junctions. By further introducing additional thiohexyl (SHe) anchors to the TTF units, we develop molecular wires with multiple binding sites and demonstrate reversibly switchable electrode-molecule contacts and junction conductance through mechanical control. These findings show a superb electrode-molecule interface and provide a new strategy for precisely tunning the conductance of molecular devices towards new functions.

## Abstract (web summary)

Though charge transport in molecular devices depend on the electrode-molecule interface, controlling the interface’s contact geometry remains a challenge. Here, the authors report molecular junctions featuring TTF-fused NDI molecules anchored to gold electrodes through direct TTF-Au contacts.

## Introduction

Charge transport across electrode-molecule interface plays significant roles in determining the properties and functions of molecular electronic devices^1–4^. Typically, terminal anchors are introduced to molecular bridges to form electrode-molecule contacts and modify the interfacial interactions and structures^5–7^. For instance, amines^8^, thioethers^9,10^, and pyridines^11^ are commonly used as anchors to bind to gold electrodes through dative interactions, while thiol^12^ and alkyne^13^ anchors have been used to form covalent gold–sulfur (Au–S) and gold–carbon (Au–S) contacts. It is worth noting that, besides the nature of the electrode-molecule contacts, the orbital overlap between anchors and molecular bridges is also a key factor in determining the interfacial electronic coupling strength. In general, a metal–anchor bond that aligns with conjugated orbitals of molecular bridges can provide efficient interfacial orbital mixing and facilitates charge transport. However, it is challenging to precisely control the electrode-molecule contact geometry in molecular devices, and the misalignment between the metal–anchor bond and the conducting channel introduces additional resistance that lowers the conductance of the device.

Tetrathiafulvalene (TTF) finds important applications in organic conducting materials^14–16^, supramolecular chemistry^17,18^, and molecular machines^19,20^ due to its remarkable electron-donating ability and redox properties. TTF is also regarded as an important unit in molecular electronics since it was proposed as a building unit of the first theoretical model of the single molecular rectifier in 1974^21–24^. Since then, several TTF or π-extended tetrathiafulvalene (exTTF)-based molecular wires have been prepared by incorporating TTF or exTTF active center into the molecular backbone. In these systems, additional terminal anchors (thiol or thioether) are required to connect the TTF or exTTF molecular bridge to the electrode^25–28^. Using these anchors, however, increases the length and flexibility of the molecules, hence leading to a lower conductance. Herein, we report new TTF-fused naphthalene diimide molecular wires that can bind to gold electrodes and form single-molecule junctions through direct TTF–Au coupling in which the sulfur atoms of TTF bind to gold electrodes to form the Au–S bonds. In these junctions, the Au–S bonds are nearly coplanar with conjugated molecular orbitals. This produces strong electronic coupling at the electrode-molecule interface, which further leads to remarkable high conductance of 10^−2^ *G*_0_ over a transport length of ~1.5 nm. We further introduce additional thiohexyl anchors to the terminal TTF units by taking advantage of their chemical modification feasibility. We show that the electrode-molecule binding sites in these wires can be either TTF–Au or thiohexyl-Au modes (see below), and importantly these binding sites can be mechanically controlled in a reversible manner, yielding tunable interfacial electronic coupling and hence the conductance of the single-molecule devices. These findings demonstrate that TTF can act as both functional unit and anchor for constructing highly conducting molecular wires, and offering a new way of modulating interfacial properties of molecular devices through mechanical control.

## Results

### Synthesis of NDITTF molecules

**Fig. 1:**
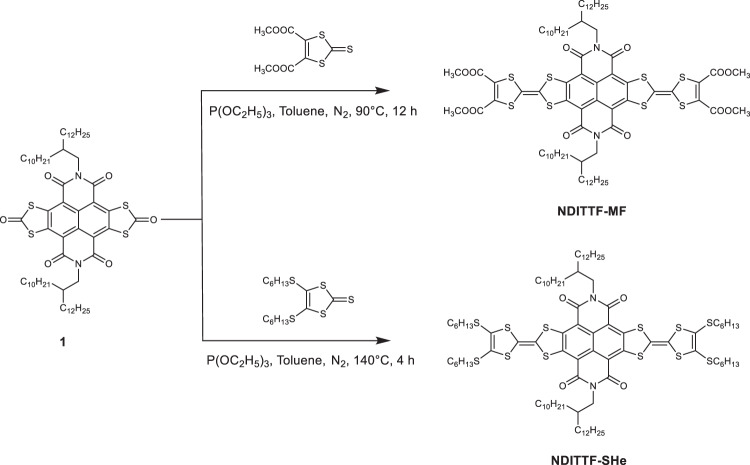
Chemical structures of NDITTF-MF and NDITTF-SHe and their synthetic approaches. Conductance measurements. We firstly probe the electronic transport properties of the four carboxylic acid methyl ester groups terminated TTF molecule (NDITTF-MF, see Fig. 1) using the scanning tunneling microscope-break junction (STM-BJ) technique (Fig. 2a), which can measure single-molecule conductance at room temperature in ambient environment^8,30^. Briefly, we repeatably drive a gold STM tip in and out of contact with a gold substrate in 1,2,4-trichlorobenzene (TCB) solution of NDITTF-MF molecule (0.01–0.1 mM). After the Au–Au point contact is broken, the molecule can bridge the two electrodes and form a single-molecule junction. We measure the conductance as a function of the tip-substrate displacement during this process. The inset of Fig. 2b shows sample conductance traces of NDITTF-MF measured at an applied tip bias of 0.5 V. A clear conductance plateau appears at ~10^−2^ *G*_0_ (where *G*_0_ = 2*e*^2^/*h* is the quantum of conductance), indicating the formation of single-molecule junctions of NDITTF-MF.

We design and synthesize TTF-based molecular wires NDITTF-MF and NDITTF-SHe (see Fig. 1) according to the procedures detailed in the Supplementary information (Supplementary Fig. 1)^29^. These wires contain an electron acceptor naphthalene diimide (NDI) core, which is flanked with two TTF units and forms a conjugated electron donor–acceptor–donor (D–A–D) framework. Various terminal substituents are incorporated into TTF units to modulate the interaction between molecules with gold electrodes. Long alkyl side chains are introduced to improve their solubilities in organic solvents. The unique D–A–D structure facilitates electron delocalization across the molecular backbone and produces long wave-length absorptions and small HOMO-LUMO gaps, as demonstrated by ultraviolet-visible (UV-vis) absorption spectroscopy and cyclic voltammetry (CV) characterizations (Supplementary Figs. 2 and 3).

**Fig. 2:**
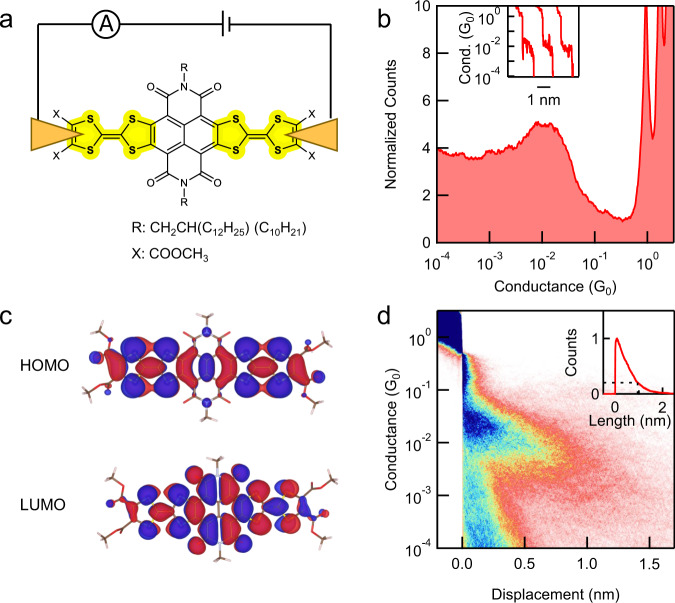
STM-BJ measurement results of NDITTF-MF. **a** Schematic of a single-NDITTF-MF molecule junction. **b** Logarithm-binned 1D histograms for NDITTF-MF measured at an applied bias voltage of 0.5 V. Inset: Sample conductance traces. **c** Gas-phase HOMO and LUMO orbitals of NDITTF-MF. **d** 2D conductance-displacement histograms for NDITTF-MF. Inset: Normalized length profiles determined from the 2D histogram. All histograms are compiled from 10,000 traces without data selection.

We repeat the STM-BJ measurements for thousands of times and compile the conductance traces into one-dimensional (1D) log-binned histograms and two-dimensional (2D) conductance-displacement histograms without any data selection. Histograms for measurements at different biases are shown in Fig. 2 and Supplementary Fig. 4. The 1D histograms show peaks around integer multiples of *G*_0_ due to the formation of point gold contacts, and a bias-independent high conductance peak at ~10^−2^ *G*_0_ corresponding to molecular junctions. Although NDITTF molecules are redox active (as shown in the CV curves in Supplementary Fig. 3), a redox process unlikely happens during the STM-BJ measurements, since an electrochemical redox reaction usually leads to a noticeable conductance shift with bias^31,32^. Further, the 2D histogram shows that the extension length of the molecular conductance plateau is ~1 nm. This indicates that the single-molecule junctions are formed across a molecule of ~1.5 nm by accounting for the ~0.5 nm Au relaxation gap upon rupture of Au contacts^33^.

To further explore the electrode-molecule interactions in these junctions, we measure the NDI based control molecule **1** in a similar environment. As can be seen from Supplementary Fig. 5, there is no well-defiened molecular conductance features, thereby ruling out a possible NDI-Au-binding mechanism. Since it is known that carboxylic acid methyl ester group typically does not bind to gold electrode, we hypothesize that the junctions are formed through the interactions between TTF and Au electrodes. Note that TTF itself does not yield clear molecular conductance features (see Supplementary Fig. 6), suggesting that the junctions are formed across the NDI backbone driven by TTF–Au interactions at the two sides of the molecules.

### Theoretical analysis

To understand the properties of single-NDITTF-MF junctions, we first perform density functional theory (DFT)-based calculations to examine its optimized molecular geometry and orbitals. Figure 2c shows the frontier orbitals HOMO and LUMO, which are most relevant to the transport in molecular devices. We see that both HOMO and LUMO are delocalized across the entire D–A–D molecular backbone. It should be noted that TTF is not aromatic, which is reflected by the boat-shaped conformation of TTF unit^34^. The orbital overlap between the S lone pairs and gold electrodes enables the formation of σ donor–acceptor S–Au bond aligned with the conducting π channel. The partial charge transfer from TTF to Au at the molecule-electrode interface promotes the transition of TTF towards a more aromatic form, producing an aromatic stabilization effect^35–39^. Contrary to other thioether anchors^9,10^, such aromatic stabilization effect provides the additional driving force for forming stable S–Au contacts. This unique effect presented in TTF not only leads to a relatively strong contact between molecule and electrode but also enables a direct orbital hybridization between Au and the delocalized conducting π-channels of the entire NDITTF backbone, yielding a strong interfacial electronic coupling and high conductance.

We next turn to DFT calculations to better understand their conducting properties using the FHI-Aims package with a Perdew-Burke-Ernzerhof (PBE) exchange-correlation functional^40–42^. The calculation details are shown in the Supplementary information. In each of the TTF unit, there are four S atoms that can in principle bind to gold electrodes. However, the outer S atoms show stronger binding abilities than the inner ones, due to the electron withdrawing effect caused by the NDI unit. Specifically, the inner S pπ orbitals can delocalize with the NDI core, whereas the outer ones are more localized, enabling them to form stronger dative contacts with the electrode. To identify the Au–S binding configurations, we model various Au–S binding modes and calculated their binding energies. As shown in Supplementary Fig. 7, the binding energy for the outer Au–S binding mode (0.56 eV) is higher than that of the inner Au–S (0.45 eV) and the Au–2S (one inner S atom and one outer S atom, 0.28 eV) bindings, suggesting that the outer Au–S binding is dominant. There might exist more complicated binding modes through multiple Au atoms and S atoms in single TTF. However, it is not straightforward to build a suitable model to simulate such bindings due to the incompatible geometries between the TTF unit and the available Au cluster models. Nevertheless, the outer Au–S binding should be the dominant scenario since this configuration (see Supplementary Fig. 8) is more in line with the measured junction length (~1.5 nm).

**Fig. 3:**
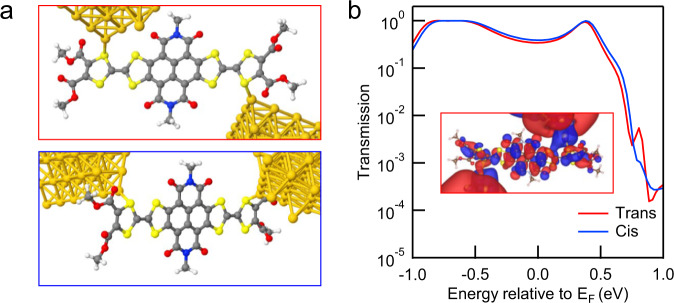
DFT-based transmission calculations of NDITTF-MF. **a** Junction structures used to compute the junction transmission functions. **b** Transmission functions against energy of the two types of junctions. Inset: Eigenstate at the Fermi energy for the junction in a *trans* conformation.

We then perform transport calculations of single-NDITTF-MF junctions. Figure 3a shows two sample junctions formed by connecting Au to the outer S atoms of TTF in a *cis* and *trans* conformation. We see that Au–S bonds are nearly perpendicular to the molecular backbone in the optimized junction geometry. This contact geometry enables the mixing of the orbitals of gold and the delocalized molecular π-orbitals, facilitating charge transport across the single-molecule junctions. Figure 3b shows that the calculated transmission spectra are almost identical for the two types of junctions. We emphasize that strong coupling between electrodes and molecular orbitals exist, as reflected by the significant peak spread of HOMO and LUMO resonances. Such strong coupling leads to remarkably high transmission probabilities (~0.5 *G*_0_) at the Fermi energy (*E*_F_). The isosurface plots of the transmitting eigenstate at *E*_F_ show a strong hybridization between gold and the delocalized molecular orbitals (see the inset of Fig. 3b), thus explaining the large interfacial coupling and high transmission probability. These calculations rationalize the observed high conductance of single-NDITTF-MF junctions at a low bias where the transport is dominated by a typical off-resonant transport mechanism.

### Conductance comparisons

We find that the peak conductance (1D histogram) of the NDITTF-MF molecule obtained at off-resonant transport regime is much higher than that of other conjugated molecular wires with similar length (Table 1). Further, we note that the molecular conductance plateau shown in the 2D histogram (Fig. 2d) is slightly sloped. This feature might be caused by the change of Au–S binding sites, or variations of molecular geometries or electrode-molecule interactions occurred during junction elongation^43,44^. We emphasize that, even though we measure the conductance near the end of the conductance plateau (see the details in the Supplementary Fig. 9), the conductance of NDITTF (10^−2.2^ *G*_0_) is still higher than most of the molecular wires shown in Table 1. These findings demonstrate that NDITTF-MF is a new core for building highly conductive molecular wires.

**Table 1:**
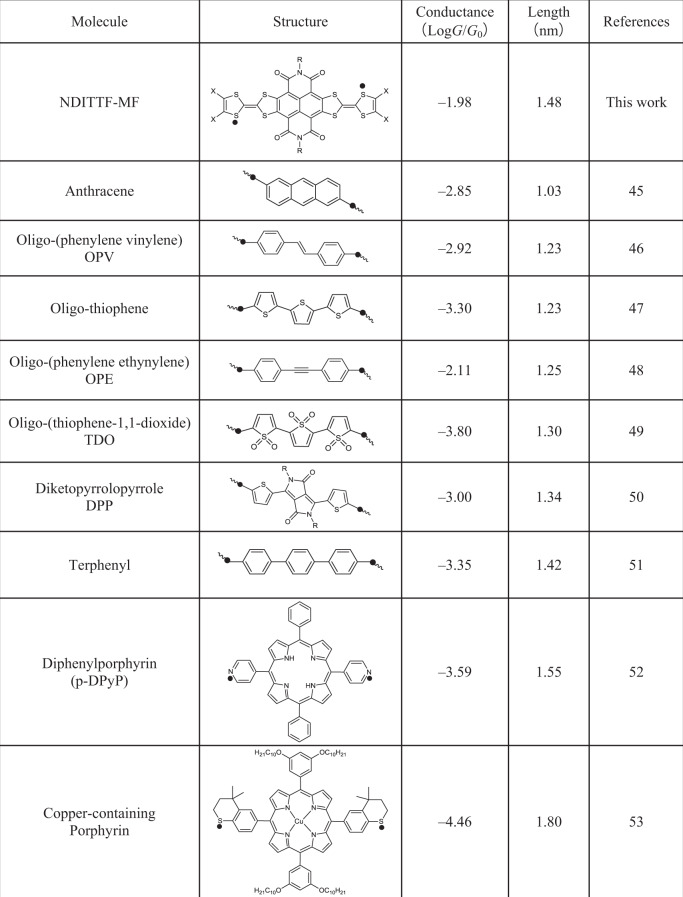
Comparison of the reported and proposed molecular wires^45–53^.

The molecular length is defined by the distance between black dots on the two sides of the molecules.

### Charge transport of thiohexyl modified NDITTF

**Fig. 4:**
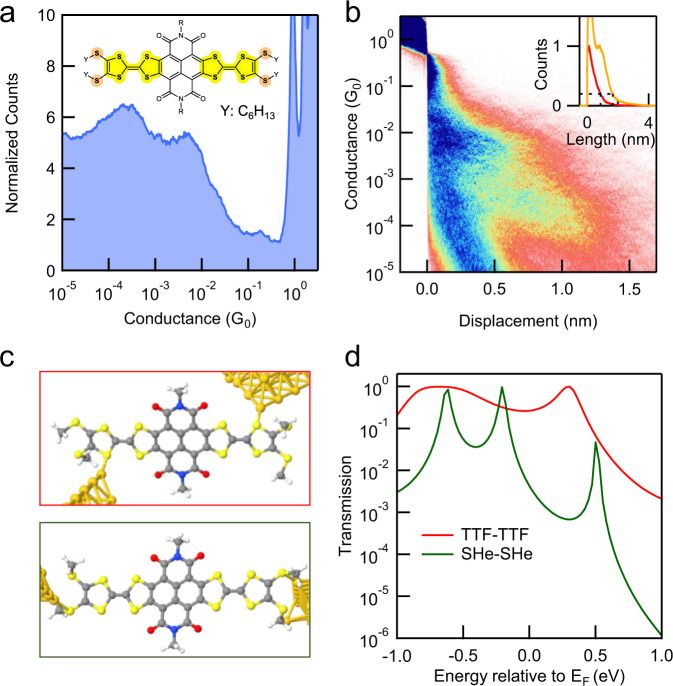
Charge transport properties of NDITTF-SHe. **a** Logarithm-binned 1D histograms for NDITTF-SHe at an applied bias of 0.5 V. Inset: Molecular structure of NDITTF-SHe. **b** 2D conductance-displacement histograms for NDITTF-SHe at an applied bias of 0.5 V. All histograms are compiled from 10,000 traces without data selection. Inset: Normalized length profiles determined from the 2D histogram. **c** Junction structures with different binding sites used to compute the junction transmission functions. **d** Transmission functions against energy of the two types of junctions.

It is known that TTF moiety can be easily substituted or modified with simple synthetic procedures^14^. By taking advantage of this feature, we design and synthesize a new TTF derivative (NDITTF-SHe, Fig. 4a) by replacing terminal carboxylic acid methyl ester groups by thiohexyls, which are common anchors used for forming dative Au–S contacts with gold electrodes (see Supplementary Information for the synthetic details). We then measured its single-molecule conductance in a similar environment using STM-BJ techniques. The 1D histograms reveal two distinct molecular conductance peaks located at ~10^−2^ *G*_0_ (High_G) and ~5 × 10^−4^ *G*_0_ (Low_G) (Fig. 4b). Figure 4c shows the corresponding 2D histograms containing two molecular conductance plateaus with different lengths. We note that the conductance values and plateau lengths of the High_G feature are in good agreement with those of the single-NDITTF-MF junctions (Supplementary Figs. 10 and 11), suggesting a similar junction structure defined by the direct TTF–Au coupling. By contrast, the Low_G conductance plateau extends much longer (~1.9 nm), indicating a longer conducting pathway across the two electrodes. We hence hypothesize that a new type of single-molecule junctions is formed through dative Au–S bonding in terminal thiohexyls.

To gain more insight of the transport properties, we calculate the molecular orbitals and transmission functions of single-NDITTF-SHe junctions with different binding scenarios (Fig. 4c and Supplementary Figs. 12–13). We first note from the HOMO and LUMO orbitals that the electron delocalization is across the NDITTF core, in agreement with that in NDITTF-MF. The calculated transmission functions are shown in Fig. 4d. We see that the junction formed through dative bonds between Au and S atoms in TTF units yields strong orbital couplings and a high transmission level at *E*_F_. This is consistent with what has been observed for the single-NDITTF-MF junctions. By contrast, much weaker couplings as reflected by the narrower resonance peaks are observed when forming junctions through terminal SHe–Au bonding. The decreased electrode-molecule coupling is attributed to the weakened orbital mixing of gold electrodes and the π orbital of the molecular backbone, which is always observed for molecular wires terminated with the similar thioether anchors^54^, and leads to a lower transmission at *E*_F_. This hence accounts for the measured lower conductance. It should be further noticed that the existence of two different conductance states in single-NDITTF-SHe junctions suggest that their conducting properties can be modulated through altering the electrode-molecule binding sites and hence the interfacial electronic coupling.

### Mechanically controlled switching measurements

**Fig. 5:**
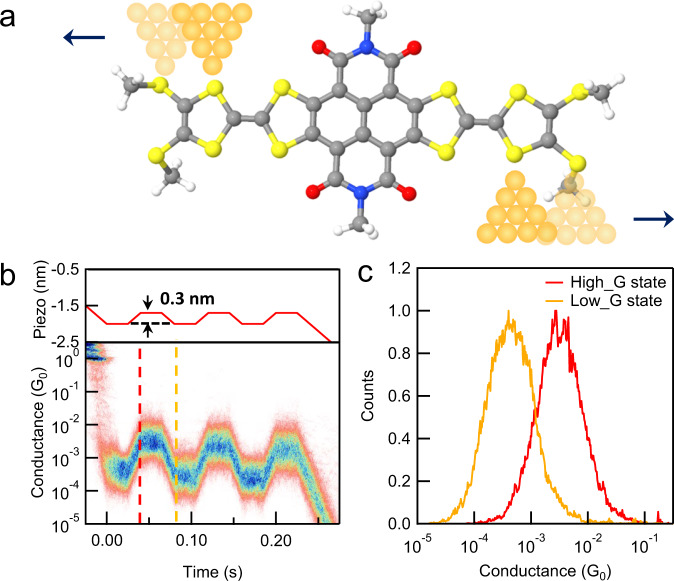
Mechanically controlled switching measurements. **a** Schematics of the single-NDITTF-SHe molecule junctions formed during the STM tip-substrate displacement modulation. **b** Piezo displacement as a function of time (top) and 2D conductance-time histograms obtained during displacement modulations. The 2D histogram is constructed from 733 traces selected out of total 20,000 traces. **c** Line profiles comparing the conductance in different regions.

Given the unique structure and conducting properties of the NDITTF-SHe, we turn to modified STM-BJ measurements to explore the mechanical tunability of the electrode-molecule coupling within individual single-NDITTF-SHe junctions (Fig. 5a). To do so, we first form a single-molecule junction, and then modulate the tip-substrate separation with an amplitude of 0.3 nm for three cycles before rupturing the junction (Fig. 5b). We obtain the 2D conductance-time histograms by aligning traces that start at a conductance corresponding to the Low_G peak shown in Fig. 4a (see Supplementary Fig. 14 for the details of data analysis). We see that the conductance reversibly switches between the Low_G and High_G states during the elongation and compression of the junctions, which is evidenced by the conductance profiles shown in Fig. 5c. We also perform the switching measurements with a piezo ramp of 0.5 nm and observe very similar conductance switching characteristics (Supplementary Fig. 15). This indicates that the switching between the Low_G and High_G state is not sensitive to the piezo ramp length, and these two conductance states are stable. Note that the binding energies of S in different positions to Au electrodes are nearly identical (0.6–0.7 eV, as shown in Supplementary Tables 1 and 2). These results thus suggest that the electrode-molecule linkages are mechanically reorganized by shifting the binding sites between the SHe and TTF S atoms (Fig. 5a), yielding the mechanical switch of interfacial electronic coupling strength and the junction conductance. Since there could be three main binding configurations, namely TTF-TTF, TTF-SHe, and SHe-SHe, one might expect three different conductance states. However, as suggested by DFT calculations (Supplementary Fig. 16), the conductance of the SHe-SHe and TTF-SHe configurations are very similar. This explains the observation that only two conductance states are present in the switching measurement.

To further validate this binding site switching mechanism, we perform similar measurements for the control molecule NDITTF-MF without SHe anchors. The results shown in Supplementary Fig. 15 suggest that this molecule only has minor conductance change compared with that of the NDITTF-SHe molecule, hence supporting the proposed switching mechanism. We emphasize that, the applied piezo ramp may not directly correspond to the length difference defined by different binding sites, since the molecular orientation might not be perfectly aligned with the tip-substrate direction. Additionally, the orientation of the molecule might change during the piezo ramp. Previously, several mechanically controlled switches have been demonstrated in molecular systems with multiple binding sites^3,55–57^. Our findings reported here provide a new design for mechanical molecular switches building on strongly coupled systems.

## Discussion

In conclusion, we demonstrate TTF to be a unique anchor that can offer strong electrode-molecule electronic coupling which is further translated into remarkably high conductance of single-molecule junctions. By introducing additional thiohexyl anchors to the TTF unit, we develop molecular wires with multiple binding sites and achieve mechanically tunable conductivity through reversibly changing the Au–S binding sites as well as interfacial couplings. This work offers new strategies to precisely modulate the electrode-molecule coupling character and sheds light for designing new functional interfaces and molecular devices.

## Methods

### STM-BJ measurements

Single-molecule conductance measurements were performed in ambient conditions and room temperature using a custom-built scanning tunneling microscope-break junction (STM-BJ) setup. During the STM-BJ measurements, we repeatably drive a gold STM tip in and out of contact with a gold substrate in 1,2,4-trichlorobenzene (TCB) solution of NDITTF molecules (0.01–0.1 mM). After the Au–Au point contact is broken, the molecule can bridge the two electrodes and form a single-molecule junction. We measure the conductance as a function of the tip-substrate displacement during this process. One-dimensional conductance histograms and the two-dimensional conductance histograms were constructed by compiling thousands of collected conductance traces.

### DFT-based calculations

We carried out density functional theory (DFT) calculations using the Perdew-Burke-Ernzerhof (PBE) exchange-correlation functional implemented by the Fritz Haber Institute ab initio molecular simulation (FHI-aims) packages. All the long alkyl side chains in the molecules were replaced by methyl groups to simplify calculation process. We first optimized the geometries of molecules to find optimal molecular structures. We then attached single Au atoms to the S anchors at the two sides of the molecules. After optimizing the geometry, two Au pyramid cluster with 60 atoms were attached to the S anchors, replacing the Au atoms used for geometry optimization. The Landauer transmission across these junctions are finally calculated using the nonequilibrium Green’s function (NEGF) formalism. The Au–S binding energies are calculated with gold pyramid clusters containing 2–7 layers of Au atoms.

## Supplementary information

Supplementary Information

## Data Availability

The main data supporting the findings of this study can be found in the manuscript and the Supplementary information file. Extra data are available from the corresponding authors (Y.Z. and G.Z.) upon request.

## Code availability

The data that support the findings was acquired using a custom instrument controlled by custom software (Igor Pro, Wavemetrics). The software is available from https://www.wavemetrics.com/.
