# Supplementary material for: Tetrathiafulvalenes as anchors for building highly conductive and mechanically tunable molecular junctions
Source: Nat Commun. 2022 Apr 4;13:1803. doi: 10.1038/s41467-022-29483-2 (PMC8980061; doi:10.1038/s41467-022-29483-2)
Supplement: Supplementary file 1 — Supplementary Information [file 41467_2022_29483_MOESM1_ESM.pdf]

Supplementary Information

Tetrathiafulvalenes as anchors for building highly conductive and mechanically tunable molecular junctions

Qi Zhou,[1,2†] Kai Song,[1†] Guanxin Zhang,[1,2*] Xuwei Song,[1,2] Junfeng Lin,[1,2] Yaping Zang,[1,2*] Deqing Zhang,[1,2] Daoben Zhu[1]

[1]Beijing National Laboratory for Molecular Sciences, CAS Key Laboratory of Organic Solids, Institute of Chemistry, Chinese Academy of Sciences, Beijing, 100190, China

[2]University of Chinese Academy of Sciences, Beijing, 100049, China

[†]These authors contributed equally to this work

E-mail: zangyaping@iccas.ac.cn; gxzhang@iccas.ac.cn

Table of Contents

Supplementary Figures

Supplementary Tables

Supplementary Methods

Supplementary Notes

Supplementary References

Supplementary Figures

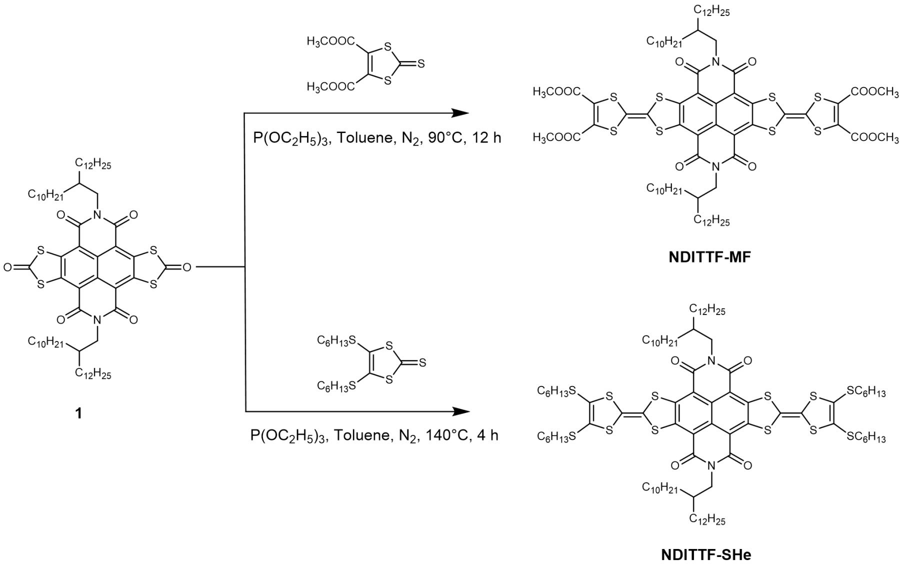

Supplementary Fig.1. Synthetic approaches. Chemical structures of NDITTF-MF and NDITTF-SHe and their synthetic approaches.

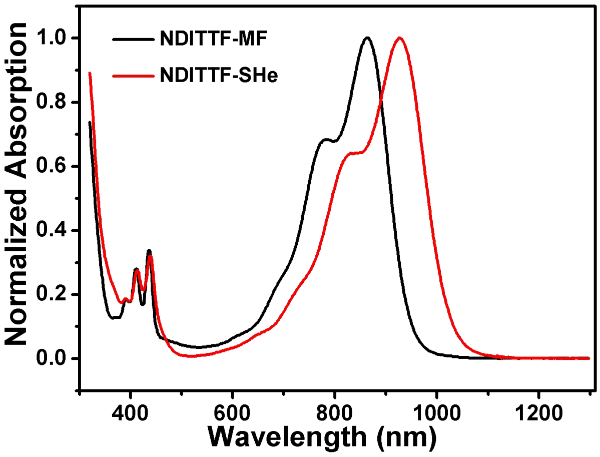

Supplementary Fig.2. Optical absorption characterizations. Normalized absorption spectra of chlorobenzene solutions of NDITTF-SHe and NDITTF-MF.

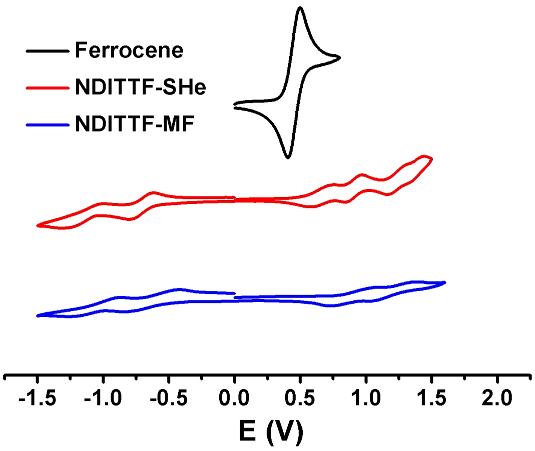

Supplementary Fig.3. Electrochemical characterizations. Cyclic voltammograms of NDITTF-SHe, NDITTF-MF and ferrocene in the mixture 1,2-dichlorobenzene and CH_2Cl_2 (1:1, *v*:*v*), respectively.

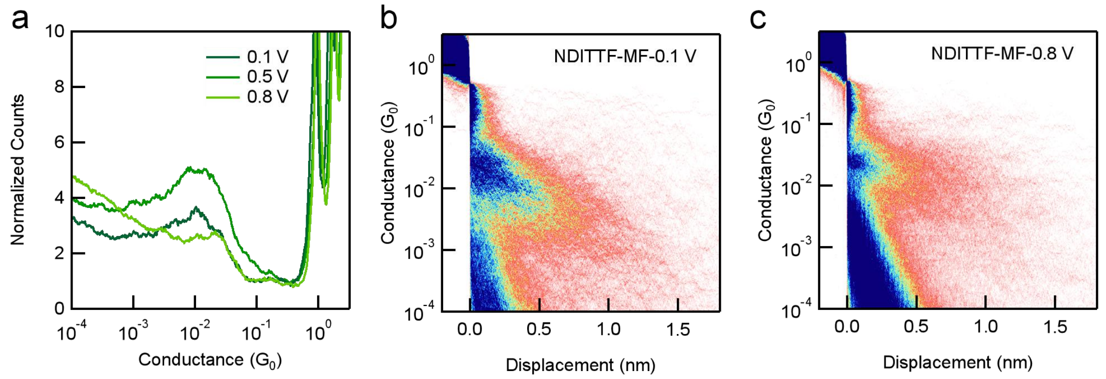

Supplementary Fig.4. Conductance histograms of NDITTF-MF. (a) Logarithm-binned 1D histograms for NDITTF-MF measured at different biases (0.1 V, 0.5 V and 0.8 V). (b-c) 2D conductance-displacement histograms for NDITTF-MF measured at the bias of 0.1 V and 0.8 V. All histograms are compiled from 10000 traces without data selection.

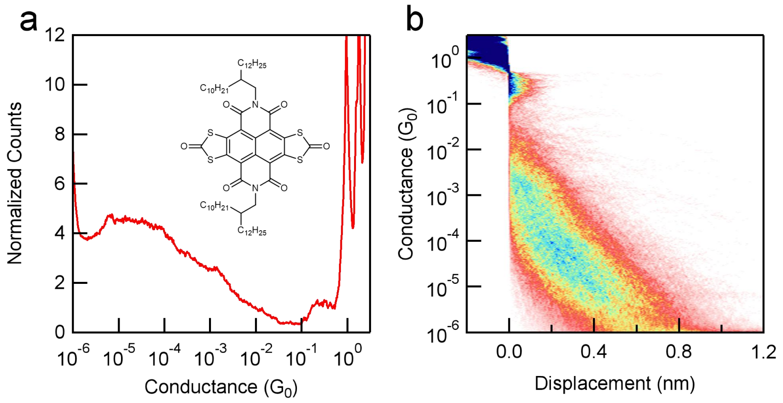

Supplementary Fig.5. Conductance histograms of control molecule 1. (a) Logarithm-binned 1D histograms for molecule 1 measured at 0.5 V. (b) 2D conductance-displacement histograms for molecule 1 measured at the bias of 0.5 V. All histograms are compiled from 3000 traces without data selection.

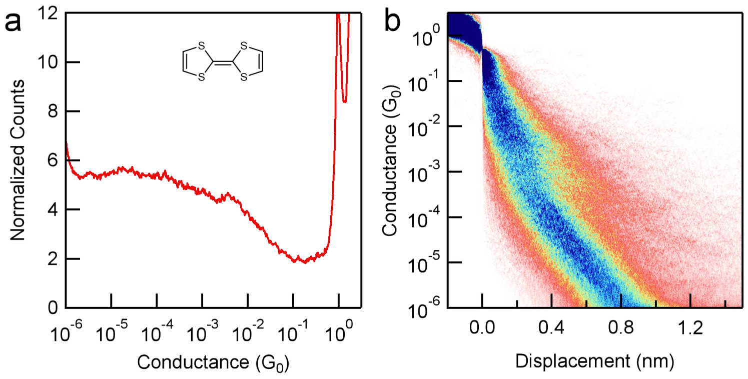

Supplementary Fig.6. Conductance histograms of control molecule TTF. (a) Logarithm-binned 1D histograms for TTF measured at applied bias of 0.5 V. (b) 2D conductance-displacement histograms for TTF measured at the bias of 0.5 V. All histograms are compiled from 3000 traces without data selection.

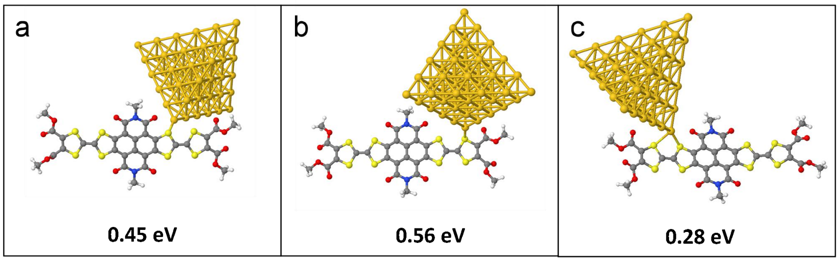

Supplementary Fig.7. Binding energies. The calculated molecular configurations and binding energies: (a) inner Au-S binding (b) outer Au-S binding, and (c) Au-2S binding.

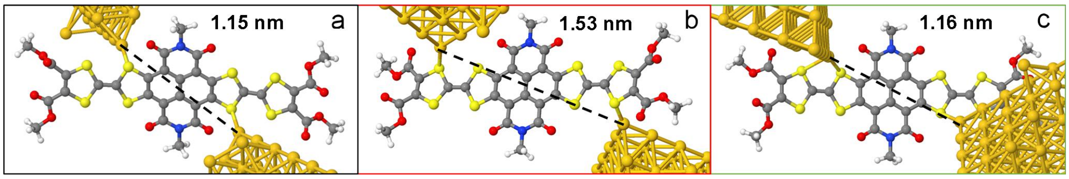

Supplementary Fig.8. Junction lengths. The calculated configurations and lengths of junctions formed with different Au-S binding sites: (a) inner Au-S binding, (b) outer Au-S binding and (c) Au-2S binding.

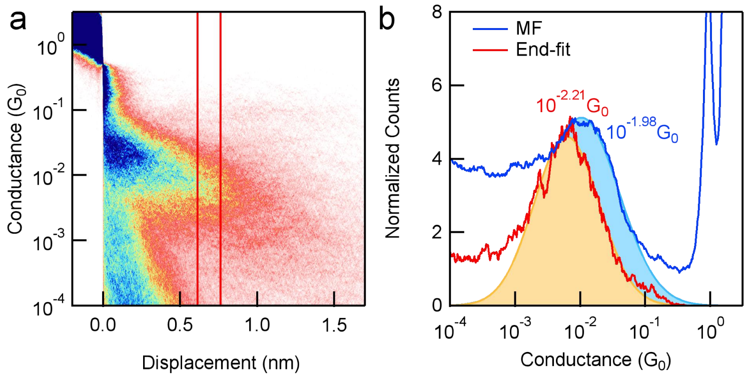

Supplementary Fig.9. Conductance histogram analysis of NDITTF-MF. (a) 2D conductance-displacement histograms for NDITTF-MF. The vertical lines near the end of the molecular feature indicate the window for determining the conductance profiles. (b) 1D conductance histograms for NDITTF-MF determined from the conductance profiles of the 2D histograms (red) and compiling the conductance traces (blue).

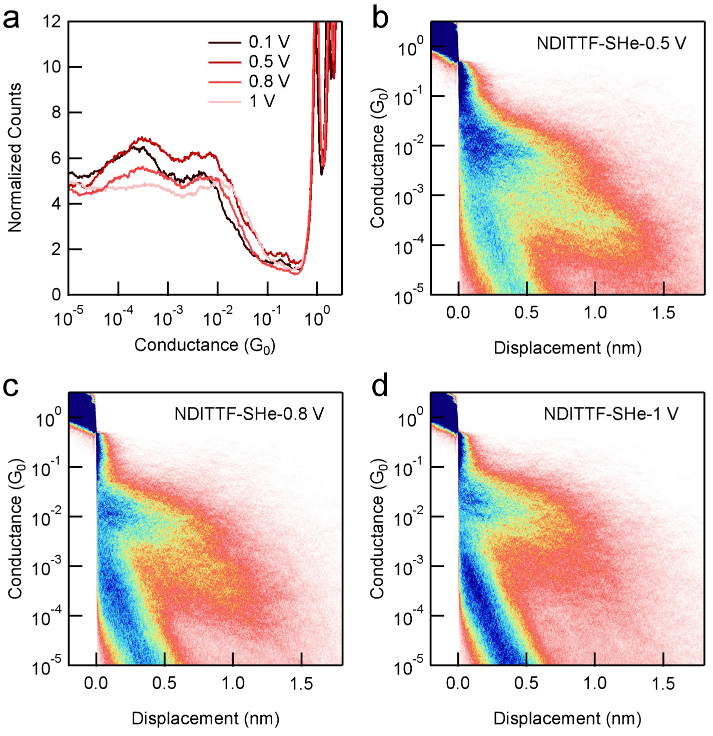

Supplementary Fig.10. Conductance histograms of NDITTF-SHe. (a) Logarithm-binned 1D conductance histograms for NDITTF-SHe measured at different biases (0.1 V, 0.5 V, 0.8 V and 1 V). (b-d) 2D conductance-displacement histograms for NDITTF-SHe measured at the bias of 0.5 V, 0.8 V and 1 V. All histograms are compiled from 10000 traces without data selection.

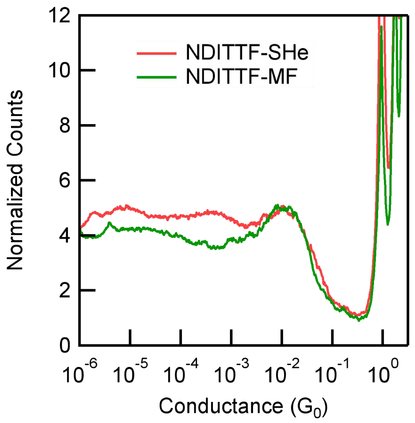

Supplementary Fig.11. Conductance histograms analysis of NDITTF molecules. Logarithm-binned 1D histograms for NDITTF-SHe at the bias of 1 V and for NDITTF-MF at the bias of 0.5 V. The comparison between two compounds at different bias shows high stability of junctions formed by TTF-Au coupling at the high bias.

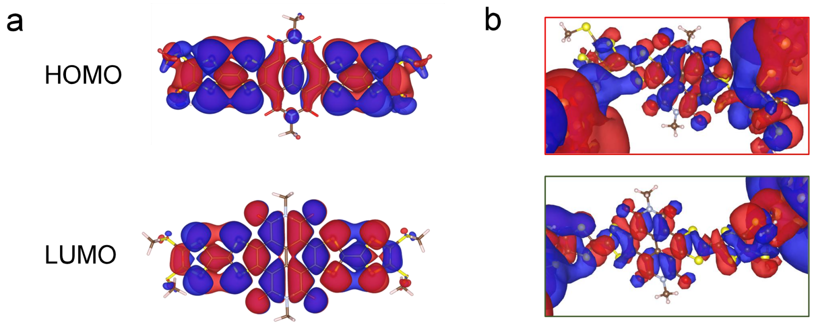

Supplementary Fig.12. Electronic structure analysis. (a) Gas-phase HOMO and LUMO orbitals of NDITTF-SHe. (b) Eigenstates at the Fermi level for the junction formed by Au-TTF coupling (red) and Au-SHe coupling (green).

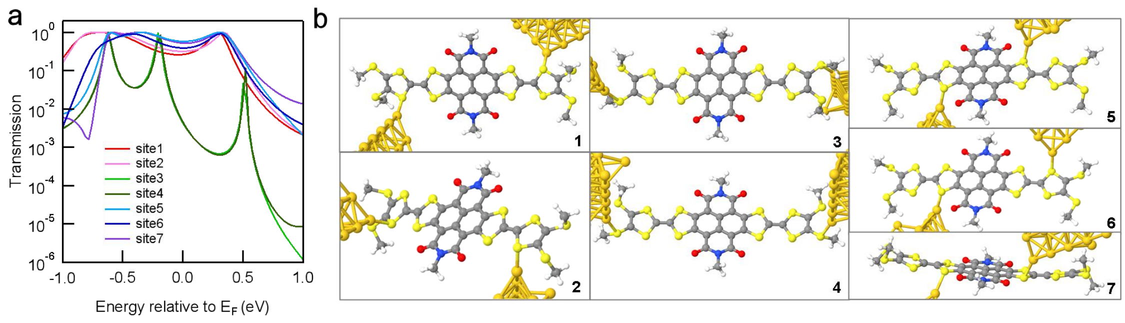

Supplementary Fig.13. Transmission calculations. (a) The calculated transmission functions against energy for single NDITTF-SHe molecule junctions with Au cluster binding to different S atoms. (b) Optimized single-NDITTF-SHe junction structures

with Au clusters binding to different S atoms.

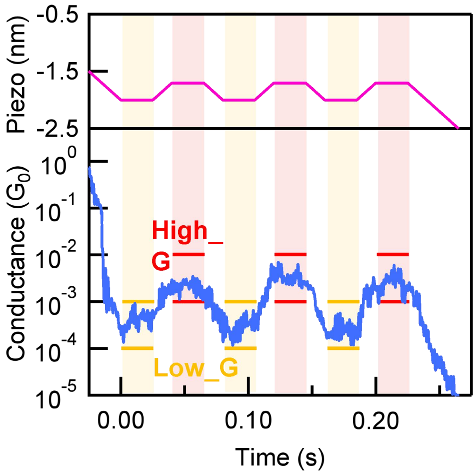

Supplementary Fig.14. Mechanically controlled switching measurement analysis. Piezo displacement as a function of time (top) and a conductance-time trace obtained during switching measurements. (Red lines define the high conductance intervals and orange lines define the low conductance intervals). To analyze the data of switching measurements, we only consider traces that show a low/high conductance switch in all of the three mechanical modulation cycles. Specially, we select traces whose conductance (in the holding region) are within certain thresholds, namely, when G_low < conductance < G_high. The intervals [G_low, G_high] illustrated by the red and orange lines are expected to contain the main conductance features, and are set to be [10^{-4} G_0, 10^{-3} G_0] and [10^{-3} G_0,10^{-2} G_0], respectively, for the Low_G and High_G conducting states (as determined from the 1D conductance histograms shown in Figure 3a)

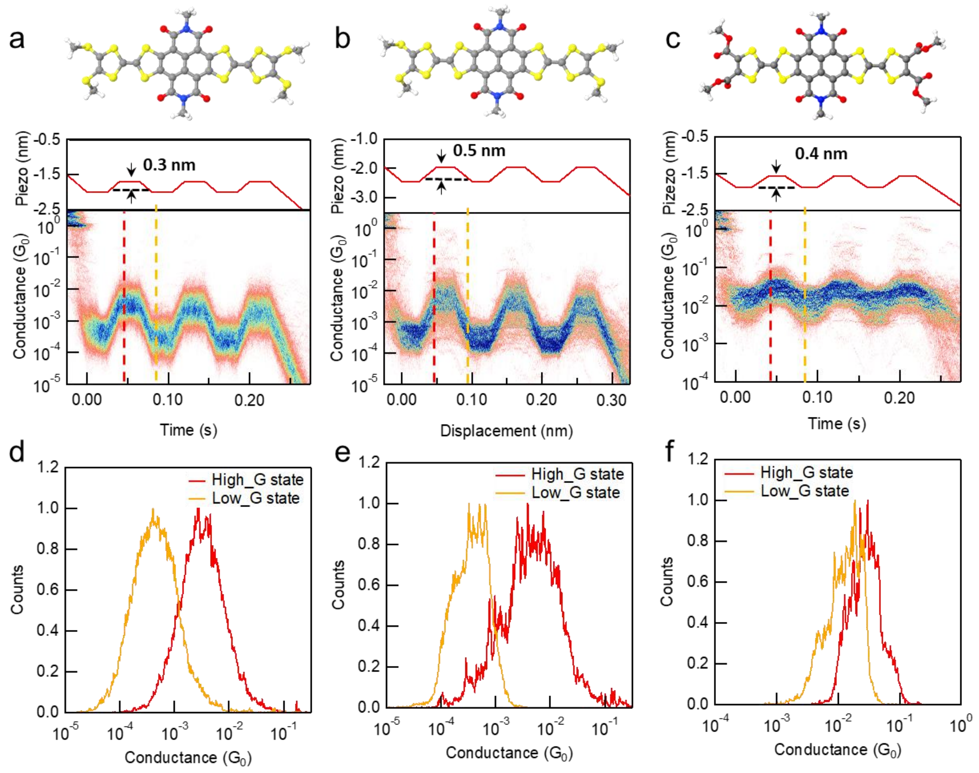

Supplementary Fig.15. Mechanically controlled switching measurement analysis. (a-c) Piezo displacement as a function of time (top) and 2D conductance-time histograms obtained during switching measurements for NDITTF-SHe and NDITTF-MF with different piezo ramp lengths. (a) The 2D histogram for NDITTF-SHe constructed from 733 traces selected out of total 20000 traces. (b) The 2D histogram for NDITTF-SHe constructed from 254 traces selected out of total 5000 traces. (c) The 2D histogram for NDITTF-MF constructed from 241 traces selected out of total 5000 traces. (d-f) Conductance profiles taken at different regions in the 2D histograms.

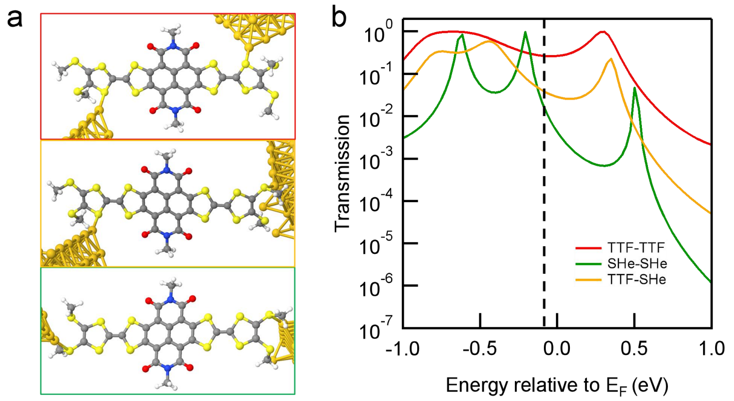

Supplementary Fig.16. Transmission calculations. (a) Junction structures with different binding sites used to compute the junction transmission functions. (b) Transmission functions against energy of the three types of junctions. Note that the accurate location of Fermi level cannot be defined in our DFT calculations, we thus offset the Fermi energy (dashed line) so that the ratio between transmission of SHe-SHe and TTF-TTF is better calibrated to experimental data.

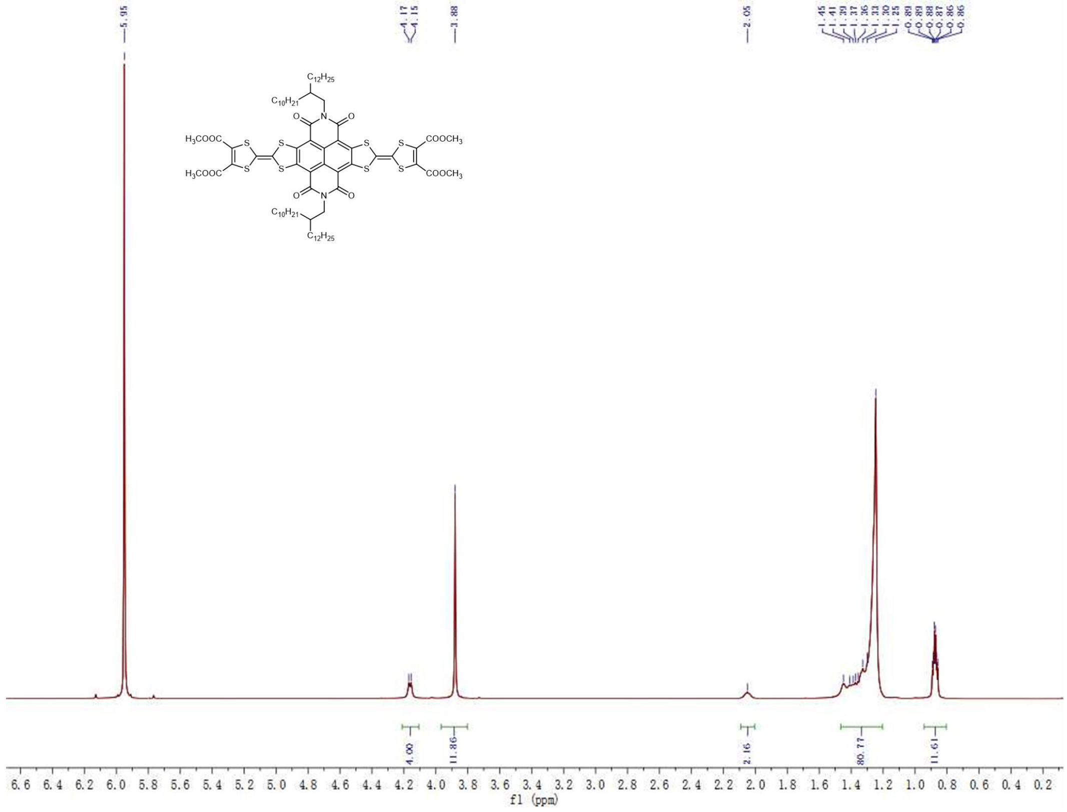

Supplementary Fig.17. NMR spectra. ^{1}H NMR spectra of NDITTF-MF in $C_6Cl_2D_4$.

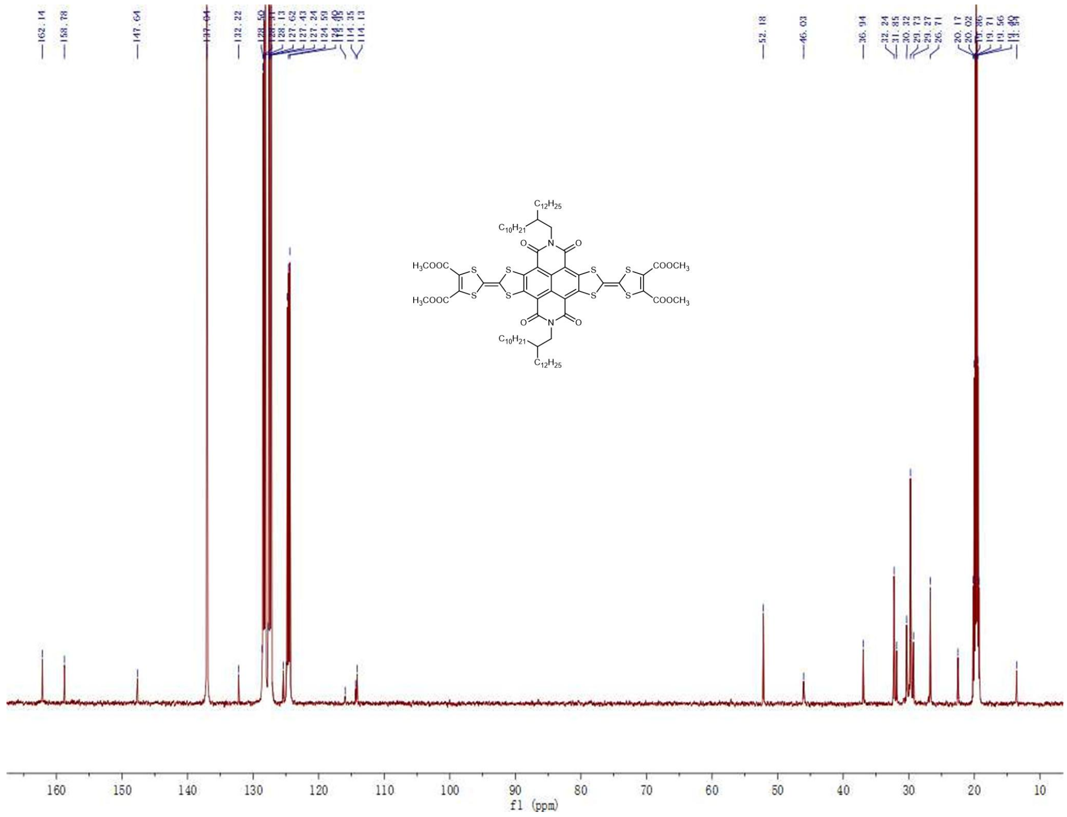

Supplementary Fig.18. NMR spectra. ^{13}C NMR spectra of NDITTF-MF in C_7D_8.

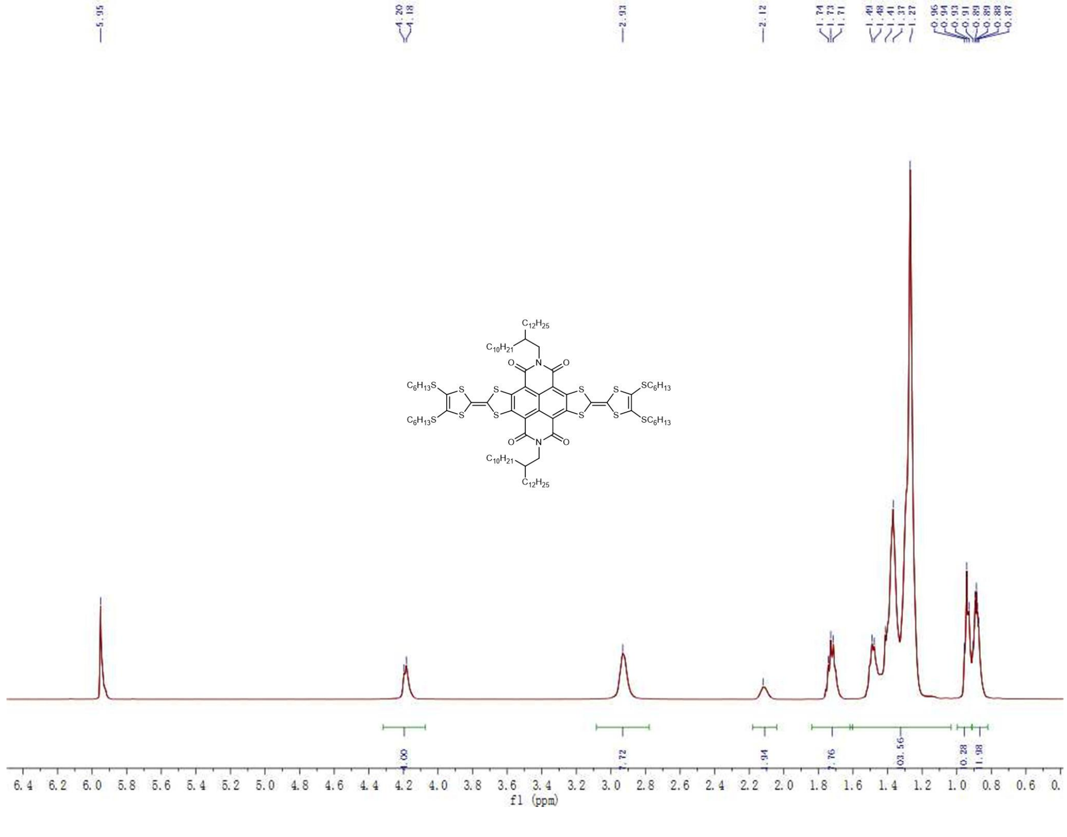

Supplementary Fig.19. NMR spectra. ^{1}H NMR spectra of NDITTF-SHe in $CDCl_2CDCl_2$.

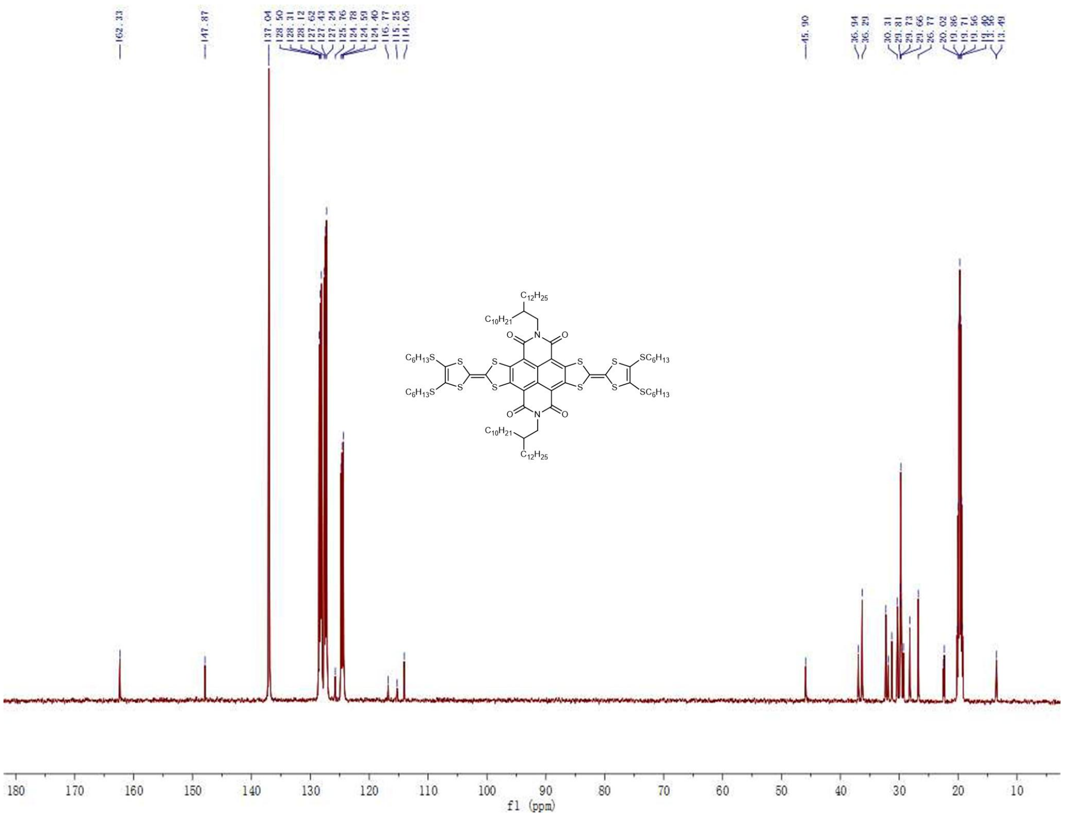

Supplementary Fig.20. NMR spectra. ^{13}C NMR spectra of NDITTF-SHe in C_7D_8.

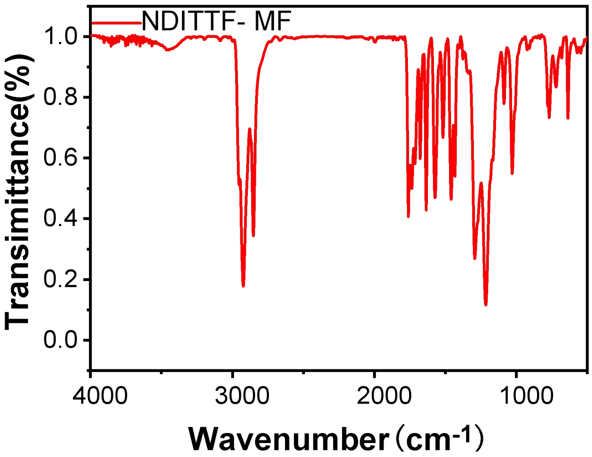

Supplementary Fig.21. FTIR spectra. IR spectra (KBr tablet) of NDITTF-MF.

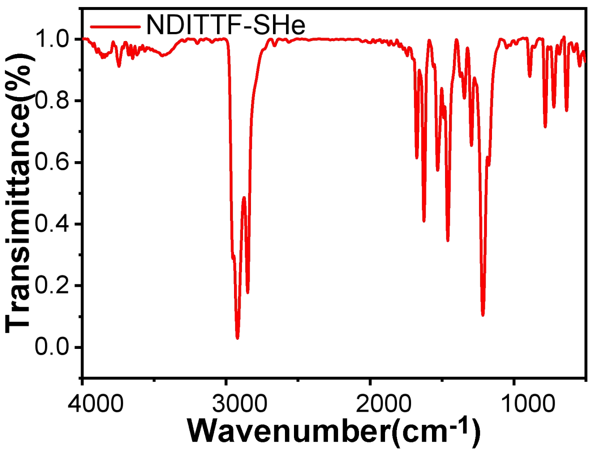

Supplementary Fig.22. FTIR spectra. IR spectra (KBr tablet) of NDITTF-SHe.

Supplementary Tables

Supplementary Table 1. Binding energies per Au-S bond calculated using Au pyramids containing 2-7 layers of Au atoms.

Layers of Au atoms	Configuration	Binding Energy (eV)
2	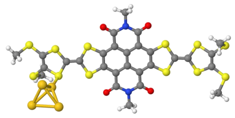	1.40
3	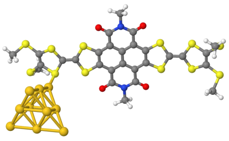	0.73
4	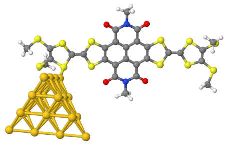	0.70
5	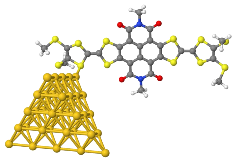	0.71
6	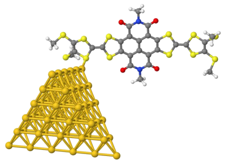	0.61
7	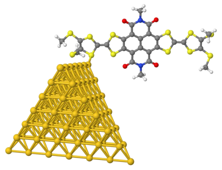	0.61

Supplementary Table 2. Binding energies per Au-S bond calculated using 6-layer Au pyramids.

Binding Sites	Binding Energy (eV)	Binding Sites	Binding Energy (eV)
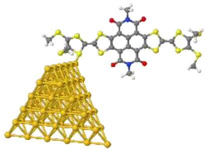	0.61	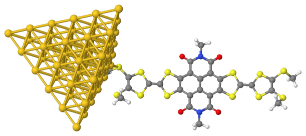	0.66
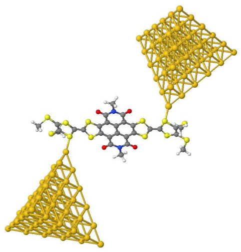	0.62	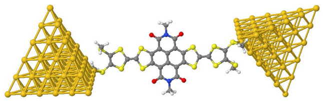	0.66

Supplementary Methods

Instrumentation. ^1H NMR and ^{13}C NMR spectra were measured on Bruker AVANCE III 500MHz spectrometer. Elemental analysis was conducted on a Carlo-Erba-1106 instrument. Matrix assisted laser desorption/ionization time-of-flight (MALDI-TOF) mass spectra were collected on a Bruker Solarix-XR high-resolution mass spectrometer. IR spectra of the solid samples (KBr tablets) in the range 400–4000 cm^{-1} were recorded on a JASCO FT/IR-480 plus Fourier transform infrared spectrometer**.** Ultraviolet-visible (UV-vis) absorption spectra were carried out on a Shimadzu UV-2600 spectrophotometer. Cyclic voltametric measurements were carried out in a three-electrode cell by using Pt/C as the working electrode, a Pt wire as auxiliary electrode, and an Ag/AgCl (saturated KCl) as reference electrode on a computer-controlled CHI660C instrument at room temperature; the scan rate was 100 mV s^{-1}, and *n*-Bu_4NPF_6 (0.1 mol/L) was used as the supporting electrolyte. For calibration, the redox potential of ferrocene/ferrocenium (Fc/Fc^+) was measured under the same conditions.

Materials. Compound **1** was synthesized according to previous report.[S1] Other chemicals and reagents were purchased from commercial sources without further purification.

Synthesis of the NDITTF-MF. Under nitrogen atmosphere, a mixture of dimethyl 1,3-dithiole-2-thione-4,5- dicarboxylate (500 mg, 2.0 mmol), compound **1** (223 mg, 0.2 mmol) and triethylphosphite (1 mL) in toluene (10 mL) was heated at 90 ℃ for 12 h. After reaction, the resulting mixture was poured into methanol (30 mL) and filtered. The precipitate was purified by silica gel chromatography with petroleum ether (60-90 °C) and CH_2Cl_2 (4:1, v/v) to give NDITTF-MF (65 mg) in 21% yield. ^1H NMR (500 MHz, $CDCl_2CDCl_2$): δ 4.16 (d, J = 7.0 Hz, 4H), 3.88 (s, 12H), 2.05 (br, 2H), 1.45–1.25 (m, 80H), 0.89–0.86 (m, 12H). ^{13}C NMR (126 MHz, C_7D_8): δ 162.14, 158.78, 147.64, 132.22, 125.38, 115.95, 114.35, 114.13, 52.18, 46.03, 36.94, 32.24, 31.85, 30.32, 29.73, 29.27, 26.71, 22.49, 13.54. IR (KBr disk, cm^{-1}): ν = 2951, 2920, 2848, 1673, 1623, 1560, 1531, 1487, 1460, 1372, 1342, 1295, 1217, 1174, 889, 858,

783, 756, 723, 682, 632, 582, 545, 503. HRMS(MALDI-TOF): *m/z* calcd. for: $C_{78}H_{110}N_2O_{12}S_8$: 1522.5824; Found: 1522.5814. Anal. calcd. for $C_{94}H_{150}N_2O_4S_{12}$: C, 61.46; H, 7.27; N, 1.84; S, 16.83; Found: C, 61.46; H, 7.27; N, 1.85; S, 17.16.

Synthesis of the NDITTF-SHe. Under nitrogen atmosphere, a mixture of 4,5-bis(hexylthio)-1,3-dithiole-2-thione (330 mg, 0.9 mmol) and compound **1** (100 mg, 0.09 mmol) in 10 mL triethyl phosphite was heated at 140 °C for 4 h. After cooling to room temperature, the resulting mixture was poured into methanol (30 mL) and filtered. The precipitate was further purified by silica gel chromatography with petroleum ether (60-90 °C) and CH_2Cl_2 (4:1, v/v) to give NDITTF-SHe (110 mg) in 70% yield. ^{1}H NMR (500 MHz, $CDCl_2CDCl_2$): δ 4.19 (d, J = 6.7 Hz, 4H), 2.93 (br, 8H), 2.12 (br, 2H), 1.84–1.71 (m, 8H), 1.49-1.27 (m, 104H), 0.94-0.82 (m, 24H).^{13}C NMR (125 MHz, C_7D_8) δ 162.33, 147.87, 125.76, 116.77, 115.25, 114.05, 45.90, 36.94, 36.29, 32.28, 31.86, 31.26, 30.31, 29.81, 29.73, 29.66, 29.28, 28.20, 26.77, 22.50, 22.35, 13.56, 13.49. IR (KBr disk, cm^{-1}): v = 2953, 2924, 2870, 2850, 1762, 1737, 1713, 1676, 1634, 1568, 1516, 1458, 1291, 1268, 1212, 1164, 1088, 1028, 781, 768, 720, 679, 636. HRMS (MALDI-TOF): *m/z* calcd. for: $C_{94}H_{150}N_2O_4S_{12}$: 1754.8244; Found: 1754.8236, Anal. calcd. for $C_{94}H_{150}N_2O_4S_{12}$: C, 64.26; H, 8.61; N, 1.59; S, 21.90; Found: C, 64.32; H, 8.49; N, 1.79; S, 21.61.

Supplementary Notes

DFT Calculations. We carry out density functional theory (DFT) calculations using the Perdew-Burke-Ernzerhof (PBE) exchange-correlation functional implemented by the Fritz Haber Institute ab initio molecular simulation (FHI-aims) packages.[S2-S4] All the long alkyl side chains in the molecules were replaced by methyl groups to simplify calculation process. We first optimize the geometries of molecules to find optimal molecular structures. We then attach single Au atoms to the S anchors at the two sides of the molecules. After optimizing the geometry, two Au pyramid cluster with 60 atoms were attached to the S anchors, replacing the Au atoms used for geometry optimization. The Landauer transmission across these junctions are finally calculated using the nonequilibrium Green's function (NEGF) formalism. The Au-S binding energies are calculated with gold pyramid clusters containing 2-7 layers of Au atoms.

Supplementary References

(S1) Chen, G. M.; Gao, C. Y., CN105461737-A (P).

(S2) Perdew, J. P.; Burke, K.; Ernzerhof, M., *Phys. Rev. Lett.* **1996**, 77, 3865.

(S3) Blum, V.; Gehrke, R.; Hanke, F.; Havu, P.; Havu, V.; Ren, X.; Reuter, K.; Scheffler, M., *Comput. Phys. Commun*. **2009**, 180, 2175.

(S4) Havu, V.; Blum, V.; Havu, P.; Scheffler, M., *J. Comput. Phys*. **2009**, 228, 8367.
